# Building Infectious Disease Research Programs to Promote Security and Enhance Collaborations with Countries of the Former Soviet Union

**DOI:** 10.3389/fpubh.2015.00271

**Published:** 2015-11-26

**Authors:** James C. Bartholomew, Andrew D. Pearson, Nils Chr. Stenseth, James W. LeDuc, David L. Hirschberg, Rita R. Colwell

**Affiliations:** ^1^Lawrence Berkeley National Laboratory, University of California Berkeley, Berkeley, CA, USA; ^2^Center for Bioinformatics and Computational Biology, University of Maryland Center for Advanced Computer Studies, University of Maryland, College Park, MD, USA; ^3^Centre for Ecological and Evolutionary Synthesis (CEES), Department of Biosciences, University of Oslo, Oslo, Norway; ^4^Galveston National Laboratory, Department of Microbiology and Immunology, University of Texas Medical Branch, Galveston, TX, USA; ^5^Department of Interdisciplinary Arts and Sciences, University of Washington Tacoma, Washington, DC, USA

**Keywords:** microbial ecology, science education, infectious diseases, collaborative research, biosurveillance, global health security, epidemiology, microbial genetics

## Abstract

Addressing the threat of infectious diseases, whether natural, the results of a laboratory accident, or a deliberate act of bioterrorism, requires no corner of the world be ignored. The mobility of infectious agents and their rapid adaptability, whether to climate change or socioeconomic drivers or both, demand the science employed to understand these processes be advanced and tailored to a country or a region, but with a global vision. In many parts of the world, largely because of economic struggles, scientific capacity has not kept pace with the need to accomplish this goal and has left these regions and hence the world vulnerable to infectious disease outbreaks. To build scientific capability in a developing region requires cooperation and participation of experienced international scientists who understand the issues and are committed to educate the next generations of young investigators in the region. These efforts need to be coupled with the understanding and resolve of local governments and international agencies to promote an aggressive science agenda. International collaborative scientific investigation of infectious diseases not only adds significantly to scientific knowledge, but it promotes health security, international trust, and long-term economic benefit to the region involved. This premise is based on the observation that the most powerful human inspiration is that which brings peoples together to work on and solve important global challenges. The republics of the former Soviet Union provide a valuable case study for the need to rebuild scientific capacity as they are located at the crossroads where many of the world’s great epidemics began. The scientific infrastructure and disease surveillance capabilities of the region suffered significant decline after the breakup of the Soviet Union. The U.S. Cooperative Threat Reduction (CTR) Program, a part of the U.S. Department of Defense, together with partner countries, have worked diligently to improve the capabilities in this region to guard against the potential future risk from especially dangerous pathogens. The dissolution of the Soviet Union left behind many scientists still working to study pathogens using antiquated protocols in unsafe laboratories. To address this situation, the CTR program began improving laboratory infrastructure, establishing biosafety and biosecurity programs, and training scientists in modern techniques, with emphasis on biosurveillance and safe containment of especially dangerous pathogens. In the Republic of Georgia, this effort culminated in the construction of a modern containment laboratory, the Richard G. Lugar Center for Public Health Research in Tbilisi to house both isolated especially dangerous pathogens as well as the research to be conducted on these agents. The need now is to utilize and sustain the investment made by CTR by establishing strong public and animal health science programs in these facilities tailored to the needs of the region and the goals for which this investment was made. A similar effort is ongoing in other former Soviet Republics. Here, we provide the analysis and recommendations of an international panel of expert scientists appointed by the Cooperative Biological Engagement Program of the Defense Threat Reduction Agency to provide advice to the stakeholders on the scientific path for the future. The emphasis is on an implementation strategy for decision makers and scientists to consider providing a sustainable biological science program in support of the One Health initiative. Opportunities, potential barriers, and lessons learned while meeting the needs of the Republic of Georgia and the Caucasus region are discussed. It is hoped that this effort will serve as a model for similar scientific needs in not only the former Soviet Union republics but also other regions challenged by infectious diseases where the CTR program operates.

## Introduction

Understanding complex factors associated with occurrence and spread of infectious diseases is of fundamental importance in promoting health security. The biological world is a complex and constantly changing landscape while significant progress has been made in identifying pathogens that cause disease and producing therapies and vaccines to counter them, the reality is that even the best efforts to mitigate a disease threat can be confounded. Lederberg commented on this phenomenon many years ago when he argued that humanity does not stand a chance against the power of microbes to develop resistance to therapies unless scientists can collaborate and combine their intellectual powers in research to understand and develop novel approaches to alleviate diseases (1). Improving the capabilities of developing regions of the world to understand and address outbreaks of infectious diseases is paramount to saving lives globally. It can add considerably to the intellectual reserve needed to address these problems and reduce the spread to other regions. While there are powerful research engines in Europe and the Western world, a thorough and deep understanding of the nature of an outbreak can often best be understood by research conducted as close to the site of the outbreak as possible. Unfortunately, much of the less well-developed world where most of the emerging infections arise lacks modern research capabilities that can provide the necessary understanding. And trying to understand environmental and cultural aspects of how an outbreak arises when working in a laboratory 3000 miles away from the outbreak is less effective and likely to take too much of the precious time needed to contain the threat.

While natural evolution of infectious agents and the emergence of new infectious diseases remain the major threat to health security, molecular engineering makes it possible to modify microorganisms with relatively few resources, and that raises the concern that terrorist groups will apply these technologies to achieve nefarious goals. Bioterrorism poses a genuine threat, especially in an unstable world. As terrorist activities increase, application of bioterrorism becomes more likely. This is a particular concern in the less developed world where the threat may be most serious. Just as it is difficult to predict exactly when the next natural infectious disease outbreak will occur, it is also difficult to predict how a bioterrorist will construct a biological weapon and where it will be used. An effective research program to address all these issues for a particular region must remain current with developments in the science of infectious diseases as well as knowledgeable about ecological pressures on the local microbial community. The common link in a program to deal with any kind of outbreak is to assemble the tools and the skills to rapidly understand the nature of the outbreak, the factors playing a role in its occurrence and spread, and therapies to counter the threat. To recognize a new outbreak, it is important to have a full understanding of the spectrum of infectious agents endemic in a region over time to provide a baseline to recognize any anomalies and for predicting future events. Such understanding requires effective biosurveillance coupled with research on those agents important for health security, both regionally and global.

The U.S. Cooperative Threat Reduction (CTR) Program has been active in many regions of the world to help reduce the threat from especially dangerous pathogens. This effort is particularly targeted to reduce the threat from biological weapons and began in Russia in 1997 following the publication of a report from the U.S. National Academy of Sciences on “Controlling Dangerous Pathogens: A Blueprint for U.S. – Russian Cooperation” (2) It is now active and effective in many of the former Soviet Union republics as well as many other countries around the world. Its progress is most advanced in the Republic of Georgia where it has built an extensive network of laboratories, trained many scientists, and promoted studies to understand the nature of pathogens endogenous to the region. The center piece of the CTR effort in the Republic of Georgia is the Richard G. Lugar Center for Public Health Research (CPHR) in Tbilisi, a modern well-equipped containment laboratory design to safely house especially dangerous pathogens and the research conducted on these agents. It was developed jointly by the U.S. and Georgian governments and constructed with funds allocated by the U.S. Department of Defense through the CTR program to provide an early warning system for occurrence and potential spread of infectious diseases originating in Georgia and impacting the rest of the world. Establishment of the new central laboratory and its associated satellite laboratories throughout the region represents a unique opportunity to provide state of the art advances in life sciences that will assist health professionals combat infectious disease pathogens of concern no matter what their origin. The system was designed to allow for studies to be conducted on identified pathogens with the utmost regard for biosafety of the workers and the community and to provide a rapid report of information to the rest of the world. With this system in place and operational, Georgia now has the laboratory system needed to meet reporting requirements under the WHO International Health Regulations (IHR) and the World Organization for Animal Health (OIE), as well as the capability to support the Global One Health Initiative.

The CPHR is not only an important resource for meeting the challenge of public and animal health improvement for Georgia and the Caucasus region but also has the potential to serve as a catalyst for science advances throughout the region. The CPHR has the potential for connecting Georgian science with the world community of scientists through a scientific program designed to support collaborations and build partnerships across the Georgian scientific community, the region, and internationally.

## The Advisory Committees

The Defense Threat Reduction Agency (DTRA) as part of the U.S. Department of Defense through CTR and its Cooperative Biological Engagement Program (CBEP) has been responsible for funding and managing much of the science that has been ongoing at the CPHR. As the construction of the CPHR was completed, CBEP recognized the need to establish an international committee of peers to provide advice and transparency for the scientific program at the CPHR and its satellite laboratories. An International Scientific Advisory Council (ISAC) was assembled in 2010 comprised largely of volunteer scientists from around the world. These scientists used their experience and training to analyze all aspects of the scientific strengths of Georgia’s existing public and animal health programs and global partnerships. The ISAC developed and submitted to CBEP a set of overarching recommendations, outlining the steps needed to ensure a sustainable scientific program for the CPHR to meets the goals of Georgia and the region as well as the CTR Program.

### International Science Advisory Council

The ISAC included internationally respected scientists each with a strong interest in promoting science in developing regions. Table 1 lists members of the ISAC. While most of the ISAC had their scientific base in Europe or the U.S., their combined experience and countries of origin spanned the globe, from South America to China. It was important that Council membership included those who, while their science was pursued in institutes located outside of Georgia, their roots and educational background were Georgian. This ensured that the Council would have the perspective of native borne Georgian scientists who were practicing their science internationally. All the scientists comprising the ISAC had considerable experience working with Georgian scientists so they already had developed mutual trust.

**Table 1:** **International Science Advisory Committee (ISAC)**.

Name	Expertise and affiliation^a^
James Bartholomew^b^, Chairman	Microbial Genetics, Lawrence Berkeley National Laboratory, University of California Berkeley, Berkeley, CA, USA
Henry M. Blumberg	Clinical and Translational Research, Emory University, Atlanta, GA, USA
Rita R. Colwell^b^	Computational Biology, Bioinformatics, and Genomics, University of Maryland, College Park, MD, USA
Carlos Del Rio	Biosurveillance, Emory AIDS International Training Program, Emory University, Atlanta, Georgia, USA
Timothy P. Endy	Infectious Disease Division, Department of Medicine, Upstate Medical University, Syracuse, NY, USA
Jason Farlow	Academic Engagement Program, Pennsylvania State University, In country scientist, Tbilisi, Republic of Georgia
Adolfo Garcia-Saestre	Department of Microbiology, Department of Medicine, Emerging Pathogens Institute, Mount Sinai School of Medicine, New York, NY, USA
Jeannette Guarner	Department of Pathology, Laboratory Medicine, Emory University, Atlanta, Georgia, USA
Tsotne Javahishvili	Department of Molecular Technologies, Ambrx Technologies, San Diego, California, USA
Paul Keim	Translational Genomics Research Institute, Northern Arizona University, Flagstaff, Arizona, USA
Teymuras Kurzchalia	Max Planck Institute for Molecular Cell Biology and Genetics, Dresden, Germany
James LeDuc^b^	Galveston National Laboratory & Department of Microbiology and Immunology, University of Texas Medical Branch, Galveston, Texas, USA
Andrew D. Pearson^b^	Epidemiologist, Computational Biology, Bioinformatics, and Genomics, University of Maryland, College Park, MD, USA
David Prangishvili	Laboratoire, Biologie Moléculaire du Gène chez les Extrêmophiles, Institut Pasteur, Paris, France
Bruno Sobral	Virginia Bioinformatics Institute, Virginia Tech, Blacksburg, Virginia
Nils Chr. Stenseth^b^	Evolutionary Biologist, Centre for Ecological and Evolutionary Synthesis (CEES), Department of Biosciences, University of Oslo, Oslo, Norway
Adrian Whatmore	Department of Bacteriology, Animal and Plant Health Agency, New Haw, Addlestone, Surrey, UK
Ruifu Yang	Institute of Microbiology and Epidemiology, Academy of Military Medical Sciences, Beijing, China

*^a^Affiliation at the time of serving on the ISAC*.

*^b^Also member of the Panel of Experts (POE)*.

### Panel of Experts

The recommendations put forth by the ISAC were high-level science and dealt primarily with many of the political steps needed to ensure a successful scientific program. To translate the ISAC recommendations into an action plan for the science, CBEP commissioned a Panel of Experts (POE) drawing on membership of ISAC and receiving the official sanction of the U.S. government. CBEP charged the POE to assess the ISAC recommendations and prepare a plan for integration and implementation of the recommended science at the CPHR. Drawing on the initial discussions of the ISAC, the POE was tasked to develop a Strategic Science Agenda (SSA) and an Implementation Plan (IP) to turn recommendations into actions to aid the Georgians utilize and sustain the resources of the CPHR and its associated laboratories.

The POE comprised James C. Bartholomew, Chairman; Rita R. Colwell, Andrew D. Pearson, Nils Chr. Stenseth, and James W. LeDuc. In 2015, David L. Hirschberg, Department of Interdisciplinary Arts and Sciences University of Washington Tacoma, joined the POE to aid in implementation of the plans for the Lugar Center. While the POE was a stand-alone committee, it used the full resources of the ISAC when necessary.

## Operating Plan of the ISAC and POE

The ISAC developed its recommendations for implementing an effective science program on infectious diseases centered at the CPHR supporting the needs of the region, by first assessing existing programs that support the public and animal health. It also looked back at the Soviet programs, the approaches taken, and the results obtained to understand the history shaping the current programs. At the same time, the ISAC considered the rapidly expanding understanding of the complexity of the biological world brought about by modern analytical techniques and how these advances could be applied to address issues unique and important to the region. It reviewed current plans for operating the CPHR and the laboratory network in Georgia, including long-term funding possibilities, staffing, training, logistics, and other resources. It met with long time collaborators on scientific project in Georgia from the U.S. Department of Defense Laboratories, the U.S. CDC, and Universities as well as scientists from international agencies with experience in Georgia to get their perspective on what was need to build a meaningful sustainable scientific program. It also discussed the issues with the U.S. Civilian Research and Development Foundation (CRDF Global) and the Shota Rustaveli National Science Foundation. Both of these organizations have extensive histories working together to support science in Georgia. These analyses were necessary to establish the base from which an appropriate future scientific program could be developed.

The most important part of the operating procedure of the ISAC was to hold extensive meetings with Georgian scientists to understand their perspective of the tasks need to develop a competitive program in health-related sciences. As part of this process, the ISAC worked with the management of the CPHR, the National Center for Disease Control and Public Health (NCDC&PH), the George Eliava Institute for Bacteriophage, Microbiology and Virology, and the Laboratory of the Ministry of Agriculture to develop a symposium entitled “Integrating Science and Public Health in Georgia.” The symposium was designed to give young Georgian investigators the opportunity to discuss their work with the ISAC and to stimulate open discussion on the current state of research on infectious diseases in the region. The symposium was held at the auditorium of NCDC&PH on March 11, 2011, and was attended by over 100 scientists from throughout the region, including representatives from the Georgian Ministries, WHO, U.S. CDC, U.S. DoD, USAID, and the U.S. Embassy. Many of the current international collaborators on Georgian research projects also were in attendance, as well as CBEP science team members. The science discussed was not only the work supported through the DTRA CBEP but also the relevant work supported by other U.S. agencies and international funding groups.

The symposium gave the ISAC the opportunity to discuss with the Georgian scientists the goals of its effort, learn from the Georgians the issues that they felt needed to be addressed, and explore solutions. After the symposium, the ISAC continued its discussions of these issues via teleconferences and email, producing a final set of overall recommendations which it submitted to the CBEP science team. These recommendations addressed not only the science that needed to be promoted but also issues dealing with how the management of the program needed to be tailored to nurture the scientific program into the future. It challenged both DTRA and the Georgian Ministries to rethink their approach to managing the activities at the CPHR and to provide the necessary resources to establish and internationally recognized science program for the region. The recommendations were applicable not only for the immediate future but also for long-term maintenance of the health security of the region. The ISAC emphasized that the science to be conducted must address infectious disease problems relevant to Georgia and the region or the program would be overshadowed by similar programs in other parts of the world. The portfolio of science recommended by the committee provided for the establishment of a wide variety of skill sets for the Georgians to deal effectively with emerging infectious diseases, whether natural, the results of laboratory accident, or a deliberate act of bioterrorism. At the same time, the projects that were selected were to address the specific infectious disease issues relevant to the Caucasus region.

The ISAC also took into account the stakeholders of the health security system that had been established in Georgia and the Caucasus region. While the Government of Georgia was the lead stakeholder, the main financial stakeholder was the United States Department of Defense through DTRA and CTR. While this investment was significant, it was not likely to continue throughout long into the future. CBEP operates in Georgia with a defined mission to counter weapons of mass destruction through modernizing the detection, reporting, and containment capabilities of the region. The ISAC concluded that for this mission to remain a success into the future it needed to be coupled with a program that had tangible benefit to the public and animal health of the region as recognized by the local governments.

International Scientific Advisory Council also recommended the CPHR establish formal relationships with international reference laboratories and scientists within international research centers conducting studies of relevance to the mission of the CPHR. Such collaborative relationships would aid in linking the CPHR program with international partners and integrate findings of studies undertaken at the CPHR into those conducted around the world. To provide an economic driver, the ISAC recommended that CPHR research activities be connected to an effort to incubate new biotechnology enterprises in Georgia and be colocated with a Biotechnology Center or “Farm.” This would be spearheaded by a focus group composed of international biotechnology industry experts and provide long-term career possibilities for Georgian scientists as well a manufacturing site for products to be delivered to Georgian and regional markets.

Central to the ISAC recommendation is that core funding be available to allow for implementation of the joint programs that were recommended and to establish competence to conduct studies of relevance to international and national funding sources. The CPHR management will need to build communications with international funding agencies early so that, as the program matures, the accomplishments of the Georgian program would be recognized by the world community of scientists. This effort can be aided greatly with the help of internationally recognized scientist such as those that make up the ISAC and POE.

## Development of a Strategic Science Agenda and Implementation Plan

The work to translate the recommendations of the ISAC into actionable projects to be conducted at the CPHR was the responsibility of the POE. The POE continued the discussions initiated by the ISAC and worked closely with the Georgian scientists to develop specific recommendations for the details of the science needed in the program. The effort included frequent trips to Georgia, as well as “kitchen table” discussions in Washington, DC, USA, teleconferences, and email. The team also realized that a complete understanding of the Georgian scientific situation required more than discussions across a meeting table or through a computer screen, and so it worked side-by-side with Georgian scientists to develop ideas including accompanying them on field trips for sample collection at sites within Georgia. The POE continuously encouraged the Georgian scientists to identify questions they believed needed to be addressed and the hypotheses they had developed to solve important infectious disease issues in the region. Throughout the process, the POE’s approach to formulating their recommendations was to listen closely to the Georgian scientists, observe, and learn what the Georgian scientists needed to bring about a successful science program. The IP developed by the POE was a direct result of integrating all these ideas and coupling them to the POE’s own experiences through the years in building and maintaining scientific programs at their home institutions.

The POE recognized that a scientific program that was not focused on issues important for Georgia and the region would soon be judged irrelevant and unsustainable. Central to this blueprint was a science plan that provided attainable goals that would build research momentum for Georgian scientists while providing important new information to the world’s scientific community. Important were recommendations to energize young Georgians to pursue science at home to improve the health and welfare of the region. Young Georgian scientists must be encouraged to undertake the training and achieve the confidence needed to compete globally in international science at the very highest level by initiating international collaborations consonant with the rapid developments that are occurring in the biological sciences today.

The SSA was developed to emulate the concept of One Health Initiative (3) for the region by developing research projects that bring animal health professionals and human health professionals to the same laboratory working on collaborative projects on the study of ecological factors playing a role in pathogen evolution. It also strongly supports the need for an integrated multidisciplinary approach to solving infectious disease questions. As such, it builds off the existing programs at the CPHR, but takes them to the next level where they can answer truly new questions about infectious diseases while it focuses on those uniquely important factors of relevance to Georgia and the region. This includes knowledge of the ecology of disease causing microorganisms with the fundamental goal to inform all health professionals in the region.

## Strategic Science Agenda

The POE recognized that the science proposed in the SSA had to address significant problems identified by surveillance data that were already available in the region and by CPHR research that was being supported by CBEP. The SSA needed to address the health needs of the region as a first priority with a base broad enough to allow it to address more global issues. Furthermore, the SSA called for enhancing the link between biosurveillance and fundamental research so as to take the studies of microbial ecology and evolution to a new level and be a model for similar studies in other regions. Skills developed in these studies would allow for rapid application to any future outbreak regardless of its source. Also clear was that all activities must be conducted following internationally approved protocols, and these protocols must be appropriate for agents in the environment of Georgia. Long-term sustainability is dependent on recognition of the importance of problems addressed, quality of data collected, and relevance both to the region and the world.

The POE called for the investigators conducting work at the CPHR to address the complexity of the microbial world and to utilize assays relevant to the problems of concern. While classical phenotypic-based assays have limitations with respect to sensitivity, accuracy, and safety, their results are still considered meaningful (4); molecular assays are highly discriminatory but must be validated and shown to be clearly related to the pathogenic phenotype (5). The array of markers that define pathogenicity is complex and not completely understood, but it is clear that any one marker cannot determine biological activity of the agent. The SSA calls for selecting appropriate assays based on a thorough knowledge of the pathogens relevant to the local situation.

**Figure 1:**
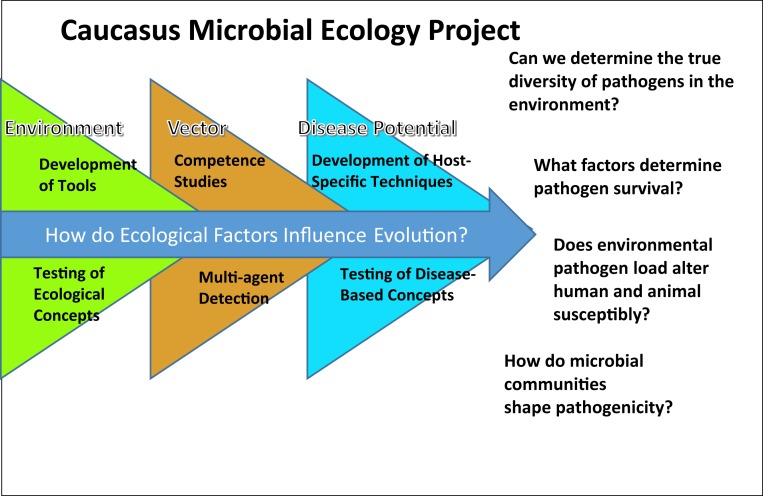
**Microbial ecology and evolution of pathogens in the Caucasus – Study Plan**.

The fundamental element in the SSA that focused all the proposed science is the proposal for the Caucacus Microbial Ecology Project (CMEP; Figure 1), studies to understand the complex microbial ecology and evolution related to pathogens of concern to Georgia and the region. CMEP would leverage off many of the existing science projects being conducted at the CPHR supported by CBEP, including the nucleic acid sequencing and annotation efforts, the Geographic Information System (GIS) mapping of pathogens, and strain characterization efforts, but would add a complex multifactorial aspect to the work to address the ecological parameters that shape changes to infectious agents. New skills would be added to the program at the CPHR including a more refined analysis of data, hypothesis generation, and the creative design of approaches to answer important question. The scientists of the CPHR would be encouraged to develop high-resolution studies to map the baseline occurrence of pathogens in the environment related to the occurrence of disease in human beings and animals. Studies were proposed to determine how complex microflora can affect activities of specific pathogens and their potential interactions with and in their host, reservoir, and vector. Recognizing that the environment represents a very extensive microbial “reservoir,” the POE recommended developing and utilizing assays to distinguish pseudo-pathogens and those relevant to diseases in the region. The ultimate goal is to provide a risk analysis of exposure to pathogens, as well as recommendations for a mitigation strategy. Of particular concern was to build projects that strengthen the working relationship between scientists studying zoonotic infectious diseases. It was clear that without a strong partnership between animal health experts and human health experts, the One Health Initiative would fail. The knowledge gained from these studies would allow understanding the pathogen spectrum of the region, namely what is there now and how, or if, it is changing, so that introduction of a new agent could be recognized. Understanding the situation in the Caucasus region would provide information that could be compared and integrated into similar studies conducted in other parts of the world, thereby achieving a global perspective.

### Linking Biosurveillance and Fundamental Research

The SSA contained several recommendations to assist the development of a biosurveillance program informed by a regional specific research agenda.

- Develop high-resolution epidemiological typing systems for regionally relevant pathogens that is based on internationally recognized methods that allow comparison of local data with those of global isolates.
- Devise schema to improve coordination and resources utilization of existing infectious disease programs sponsored by Georgia and/or international agencies. This will provide a basis for a “One Health Initiative” in Georgia.
- Establish a national plan to improve laboratory diagnostics for the region, notably strengthen coordination of surveillance for human and animal infectious disease agents that pose public and animal health problems.
- Develop an overall integrated disease reporting system for Georgia applicable to region-significant infectious diseases that is based on syndromic surveillance and coupled to the enhanced laboratory capacity of the CPHR.
- Improve diagnostic capabilities based on biological activities relevant to pathogenicity and baseline disease knowledge. This will promote implementation of relevant disease control strategies as appropriate to the region.
- Include surveillance data on pathogen near relatives to assess impact on host susceptibility.
- Establish a well-documented sample and data repository to allow reach back to samples for historical analysis, based on current understanding of relevant problems.
- Develop a knowledge-based risk benefit analysis to deal with infectious disease issues and allow efficient application of resources.
- Study the interplay of different geographical factors and how they influencing agent distribution. For example, the geographical distribution of the use of antibiotics and the appearance of resistant microbes.
- Understand the relationship of the landscape distribution between different agents.
- Determine the distribution of susceptible populations and vectors, and the factors controlling susceptibility.
- Apply statistical modeling of historical data and relevant factors determined to be functioning in the current environment.
- Study the factors influencing clonal distribution of pathogens in the environment.
- Study of genetic varieties of a given pathogen or multiple pathogens and whether they exclude one another or enhance occurrence in the region? Determine the factors that drive these relationships.
- Study of near relatives of pathogens and their role in stimulating natural immunity.
- Study the role of immunological enhancement of one version of a pathogen on another to modulate activity of pathogens of importance to the region.

### Topic Areas for Fundamental Research

The SSA called out specific fundamental research areas to be contained in the CMEP. These topic areas were select based not only on their relevance to the focus of microbial ecology but also because the skills developed during studies in these areas would transcend many other research projects of relevance to the mission of the CPHR. Studies in these areas would add capabilities to existing expertise in strain characterization and genomics being developed by CBEP at the laboratory. Another concern in selecting these topic areas was to provide opportunities to develop projects that supported and involved scientists from both the public health sectors as well as animal health scientists with a bridge to the phage research at the Eliava Institute, which is a unique and strong program in Georgia.

1. Antibiotic-resistant pathogens – detection and genomic characterization of multiple drug-resistant agents; auditing national antibiotic usage in people, animals, and agriculture; analysis of outcomes of antibiotic usage correlated with bacterial resistance patterns.
2. Vector-borne diseases – epidemiological analysis of human and environmental pathogens linked with vector-borne diseases, notably exploration of hypotheses for the molecular basis of environmental maintenance of disease agent foci in Georgia, including infections with multiple pathogens and investigation of prevalence and impact of multiple pathogens transmitted by single vectors.
3. Pathogen migration – molecular epidemiology of avian influenza linked to animal/avian borne human cases to address evolution of sequences of these viruses in the region and relationship to influenza sequences of viruses globally.
4. Zoonotic risk assessment – impact of zoonotic brucellosis on human health and veterinary practice: assessment of health gain from potential prevention strategies.
5. Food and water-borne pathogens – impact of zoonotic salmonellosis on human health and veterinary practice.
6. Horizontal gene transfer – nucleotide sequence and annotation of bacteriophages in the Eliava collection, with emphasis on horizontal transfer of genes in the evolution of pathogens.

These topics circumscribe important capabilities either already present or needed at the CPHR and associated satellite laboratories to support public and animal health in a twenty-first century research facility. The portfolio of topics and the derived projects will build relevant technical capabilities and practical confidence in Georgian science. To ensure successful implementation of the plan, scientific partners would be sought to assist in applying lessons learned from published scientific research in international settings that was carried out in programs that have relevance for Georgia. This will provide Georgian scientists with an opportunity to learn new methods, refine scientific skills in an international arena, and obtain relevant information for their research, as well as inform decision makers in Georgian Government agencies.

The SSA was submitted to the management of the Lugar Center and the CBEP science team in November, 2012, and was approved.

## The Implementation Plan

Cooperative Biological Engagement Program and the POE realized that for the SSA to be useful, it needed an effective IP to serve as a catalyst for action. The POE relied heavily on its own experience with what it takes to carry out successful completive research to develop the IP. It takes a critical mass of discussion amongst knowledge scientists addressing similar problems to push research in new directions. It takes a vision to focus the research and provide a goal to be achieved. Coupled with this is that it takes persistence in the effort to obtain funding, as the road to receiving the funding is filled with many setbacks. These setbacks need to be used as lessons learned to improve proposals for another try. It takes support from other scientists both locally and internationally as well as support from home institutes and governments who understand that quality science requires a period of incubation.

**Figure 2:**
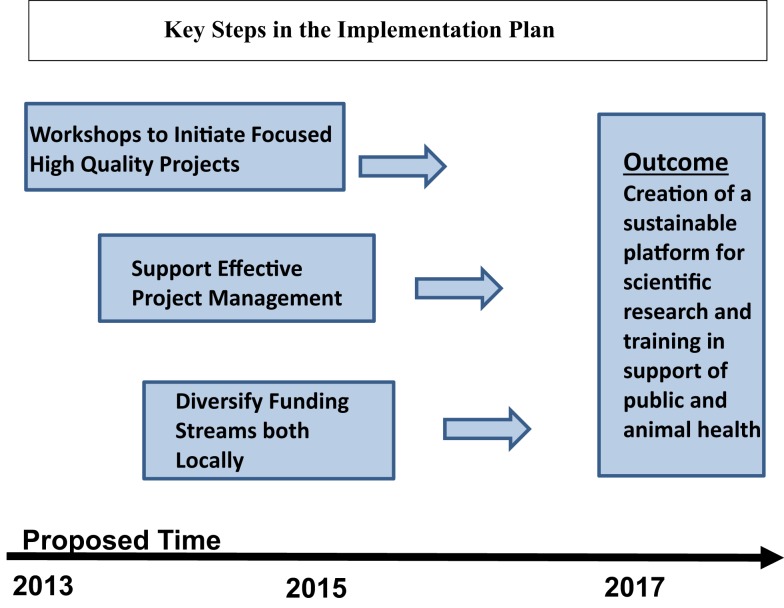
**Steps in the implementation plan**. The raw material for a successful science agenda is a set of high-quality research projects that excite both investigators and the scientific community that is strongly supported by institute management.

Figure 2 outlines the key steps in the IP. The plan is made up of steps to stimulate research ideas, plans to train in the unique aspects of managing research projects, and steps to assist in developing long-term funding to support the work of the SSA. Along the way new contacts are made with recognized scientists in areas relevant to program in Georgia.

As called out in the IP, the POE is to conduct scientific workshops on topic areas called out in the SSA. The goal is to bring a number of highly respected international scientists to Tbilisi to present results from their own studies conducted in their laboratories. The idea was to stimulate discussions with the Georgian scientists on “state of the art” research and how it might fit into future work at the CPHR. The workshops were to be hosted by Tbilisi State Medical University (TSMU) and by NCDC&PH and be designed to allow for plenty of discussion. It was through these workshops that the POE hoped to generate new ideas to serve as the focus of new proposals from the Georgian scientists in collaboration with international partners. It was hoped this would be an educational experience for both sides – the Georgian scientists would hear the work ongoing in their fields in international laboratories, and the invited scientists would see the possibilities of collaborative work in Georgia and the region.

### Workshops Progress

The POE, with the help of the Georgian Institutes, the U.S. Embassy in Georgia, and contractors for the CBEP began organizing workshops focused on the six research topic areas listed in the SSA. The workshops included Georgian scientists and international experts, with a sharing of experience and lessons learned with specific hypotheses to be tested. Workshops conducted to date have led to development of 18 competitive proposals for projects. Each workshop has included productive discussion leading to establishment of objectives for each topic area and appropriate hypotheses to be tested that suit the needs of the Georgian public and animal health objectives.

Two workshops have been held and jointly hosted by TSMU and the NCDC&PH. Both workshops were opened by the United States Ambassador to Georgia, the Rector of the University, and the Director of NCDC&PH.

The first workshop, International Workshop on Antimicrobial Resistance (AMR), was held on July 2–4, 2013. More than 200 regional scientists and scientists from countries of the former Soviet Union attended. At this first workshop, Memoranda of Understanding (MOU) to committing parties to work together to develop biological sciences in Georgia were signed by officials of TSMU, NCDC&PH, and POE members representing the University of Maryland, United States, and the University of Oslo, Norway.

As shown in Table 2, speakers were from many different countries representing different perspectives, but all focused on solving local AMR problems in Georgia as part of a global effort. The POE designed the workshops to bring together scientists from countries outside the former Soviet Union and to reunite scientists from within the former Soviet Union to stimulate a transparent environment for research, especially at the CPHR.

**Table 2:** **International Workshop on Antimicrobial Resistance (AMR)**.

Workshop #1		

Speaker	Affiliation	Title
Fred Tenover	Stanford University and Cepheid, Palo Alto, CA, USA	Use of Appropriate Technologies for the Rapid Diagnosis and Surveillance of Antimicrobial Resistance in Georgia and the Caucasus
Bruce R. Levin	Emory University, Atlanta, GA, USA	Role of Microbial Pathogen Population Dynamics in the Spread of Antimicrobial Resistance
Martin F. Polz	Massachusetts Institute of Technology, Cambridge, MA, USA	Ecological Populations of Bacteria Act as Socially Cohesive Units of Antibiotic Production and Resistance
Nadezhda Fursova	State Research Center for Applied Microbiology and Biotechnology, Obolensk, Russia	The Novel CTX-M-116 β-lactamase Gene Discovered in Proteus mirabilis is Composed of Parts of the CTX-M-22 and CTX-M-23 Genes
Tomi Kostyanev	Laboratory of Medical Microbiology, University of Antwerp, Belgium	European AMR COMBACT LAB Network
George Kamkamidze and Nino Macharashvili	Richard G. Lugar Center for Public Health Research, Tbilisi, Republic of Georgia	Gram Negative Infections in Hospital and Community Patients
Mikeljon Nikolich	WRAIR, WHO & U.S.A. CDC	AMR Surveillance in Georgia – summary of current and proposed activity: scope of the project team proposal for surveillance and research
Giorgi Chakhunashvili	National Center for Disease Control & Public Health, Tbilisi, Republic of Georgia	AMR in Georgia
Ekaterine Zangaladze	National Center for Disease Control & Public Health, Tbilisi, Republic of Georgia	MDR and XDR TB infection surveillance and control in Georgia
Rezo Adamia	George Eliava Institute of Bacteriophage, Microbiology and Virology, Tbilisi, Republic of Georgia	Bacteriophages as Potential New Therapeutics to Replace or Supplement Antibiotics
Rita Colwell	University of Maryland Center for Bioinformatics and Computational Biology, College Park, MD, USA	Steps to move forward to address AMR from an ecological and molecular genetic perspective

From this workshop, Georgian scientists and international experts developed and proposed a set of 18 competitive proposals with Georgian investigators as Principle Investigators. The POE reviewed 18 proposals and selected the top five, recommending these to CBEP for their consideration for funding through DTRA’s Basic and Fundamental Research Broad Area Announcement. The CBEP science team and reviewers considered all five of the proposals and selected the one which described a regional effort to assess occurrence of carbapenem-resistant bacteria circulating in the region for funding and recommended the proposal to DoD Policy for approval. Unfortunately, DoD Policy ruled that the work described in the proposal was outside the scope of the CBEP mission and the proposal was not funded.

While this workshop did not result in proposals being funded by DTRA, it did generate excellent proposals that receive excellent scientific reviews. These proposals will be submitted to other funding agencies whose focus is on the world-wide crisis of AMR. And it provided Georgian investigators very useful experience in preparing competitive proposals to attract international funding for their research. It also emphasized and called attention to the need to develop a program for the region to counter the threat of antibiotic-resistant strains of pathogens arising from unregulated use of antibiotics that has been the practice in this region for decades.

The second POE workshop (Table 3), Microbial Ecology of Environmental Pathogens (MEEPs), was held on December 4–6, 2014, at the same venue as the first workshop. The attendance was similar to that of the workshop on AMR. Again, discussion was lively and generated many new ideas. The workshop objective was to understand how microorganisms interact with their environment, with each other, with their vectors, and with their hosts. There was an emphasis on how ecological factors of Georgia and the region shape development of new strains and/or species of environmental pathogens and how development of newly emerging infections occurs. The program highlighted presentations of both Georgian scientists and international invitees, with excellent sharing of experiences and lessons learned from molecular-based investigations into scientific question(s) surrounding health security. This workshop, as did the first workshop, focused on identifying competitive research projects reflecting the needs of Georgian science to support public and animal health. The discussions resulted in the establishment of a set of objectives for each topic that had been identified and scientific hypotheses addressing those topics. An assessment of relevant technology and expertise to deliver project goals relevant to Georgian was provided. The international team continues to work on a broad range of topics with the Georgian scientists on how the environment serves as a natural reservoir of pathogens and how microorganisms alter their genetic composition to counter threats to their survival induced by ecological pressures caused by human activity. It is intended that the outcomes of this workshop will include proposals to study ecological drivers of pathogen change and identify new tools and new approaches to mitigate risk of emerging infections and reduce the burden to public and animal health.

**Table 3:** **Microbial Ecology of Environmental Pathogens (MEEPs)**.

Workshop #2		

Speaker	Affiliation	Title
Michael J. Mahan	University of California Santa Barbara, Santa Barbara, CA, USA	Rise of the Microbes
Elisabeth Carniel	Institut Pasteur, Paris, France	Horizontal Acquisition of a Filamentous Phage Early after Y. pestis Emergence
A. Marm Kilpatrick	University of California, Santa Cruz, CA, USA	Drivers, Dynamics and Control of Emerging Vector-borne Zoonotic Diseases
Peter Hudson FRS	The Huck Institute of Life Sciences, Penn State University, PA, USA	An Ecological Perspective on Spillover and Invasion of Infectious Diseases
Gvantsa Chanturia	National Centre for Disease Control and Public Health, Tbilisi, Republic of Georgia	Review of Tularemia Ecology in Georgia
Ekaterine Khmaladze	National Centre for Disease Control and Public Health, Tbilisi, Republic of Georgia	Discovery and Further Investigation of a New Highly Divergent Orthopoxvirus in Georgia
Giorgi Babuadze	National Centre for Disease Control and Public Health, Tbilisi, Republic of Georgia	Detection, Confirmation and Phylogenetic Analysis of Crimean-Congo Hemorrhagic Fever Virus in Human and Tick Samples Obtained During 2013-2014 Outbreaks in Georgia
Anna Machabilishvili	National Centre for Disease Control & Public Health, Tbilisi, Republic of Georgia	Transmission of Zoonotic Influenza between Humans, Pigs, and Poultry
Robert Webster	Department of Infectious Diseases, St. Jude Children’s Research Hospital, Memphis, TN, USA	Perspectives on Influenza Evolution and the Role of Research
Kornelia Smalla	Julius Kühn-Institut-Federal Research Centre for Cultivated Plants, Braunschweig, Germany	Genes in Motion – Widespread dissemination of class I integron components in soils and related ecosystems as revealed by cultivation-independent analysis
Jason Farlow	Farlow Scientific Consulting, Lewiston, UT, USA	Ecological and Within-host Implications of Viral Quasispecies
David Prangishvili	Institut Pasteur, Paris, France	Viruses of the Archaea: insights into the diversity and evolution of virus-host interactions
Marina Tediashvili and Ekaterina Jaiani	George Eliava Institute of Bacteriophages, Microbiology and Virology Tbilisi, Republic of Georgia	Diversity and Predictability of Human Pathogenic Vibrios along the Georgian Coastal Zone of the Black Sea
Britt Koskella	University of Exeter, Cornwall Campus, Tremough, TR10 9EZ, UK	Understanding Bacteriophage Specificity in Natural Microbial Communities
Marina Donduashvili	Laboratory of the Ministry of Agriculture, Tbilisi, Republic of Georgia	Epidemiological and Laboratory Surveillance of CCHF in Animals in 2014
Dennis Bente	Galveston National Laboratory, University of Texas Medical Branch, Galveston, TX, USA	Pathogenesis and Transmission of Crimean-Congo hemorrhagic fever virus
Yingzi Cong	University of Texas Medical Branch, Galveston, TX, USA	The Dynamic Influence of Commensal Bacteria on the Immune Response to Pathogens

A request for proposals (RFP) derived from the second workshop has not yet been issued. However, the participants continue to engage in discussions of ideas to be developed into projects to propose when the RFP is released.

The POE continues to develop plans for additional workshops to support the SSA. While a proposal has yet to be funded, the value of the program is abundantly clear. Georgian scientists have had experience in developing their own ideas for competitive research proposals. Many of them are committed to follow through even though they have not yet been successful in receiving funds from DTRA to carry out the work. However, through the process, they have built relationships with many new scientists from around the world and the discussions that followed have generated new perspectives on ideas of how to attack the problems of infectious disease in their region. The work on these projects needs to continue to fully develop these ideas as the Georgian and regional program matures.

### Focus Groups

Success in implementing the SSA would require effective communication fostering creative hypothesis-based research. Building a communication network not only within a project team but also throughout the institution was required to share information and ideas across project boundaries to leverage expertise throughout the program. Therefore, POE proposed the focus group concept to explore core needs of the science program. The focus group concept is based on realization that a critical mass of scientists is needed to build a sustainable program. In a complex multidisciplinary program, maintaining expertise in a technical area is often sacrificed by the team’s focus on the goal. Focus groups are designed to cut across project boundaries and allow specialists to maintain their expertise while contributing to many projects and to the overall program. A focus group comprises those individuals engaged in projects in Georgia, including international infectious disease programs and investigations. A group of experts is assembled, representing disciplines needed for advising projects underway at the CPHR. This will maximize support and integration of disciplines. Microbial ecology research is best done with an interdisciplinary approach, and interdisciplinary science works best when each discipline is fostered to a high level of expertise, while communicating lessons learned across project boundaries.

**Figure 3:**
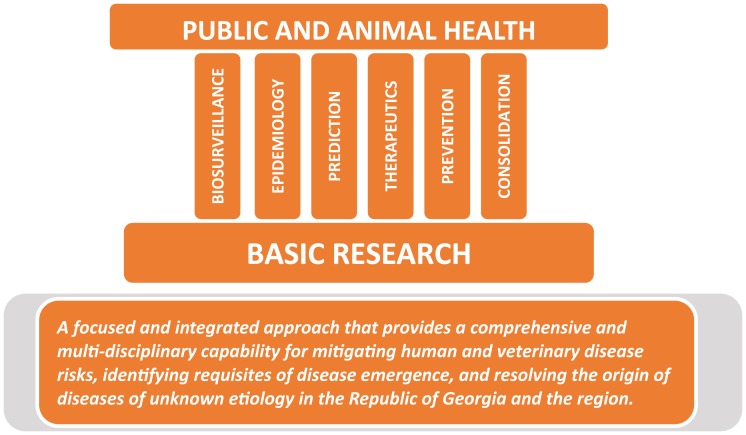
**Focus groups**. The POE Implementation Plan envisioned Focus Groups as pillars to ensure strong linkage between the needs of the public and animal health identified issues and the basic research program conducted at the CPHR enhancing the ability of the health sector to respond to disease outbreaks and providing relevance to the research program.

The focus groups would be composed of both Georgian and international scientists working as collaborators from their home institutions, or on site at the CPHR. The goal of the focus groups was to ensure the best possible approach was taken for each of the implemented projects to be achieved and that the results and ideas were shared to maximize efficiency and progress of the program as a whole. While the design of the focus groups is flexible to meet the needs of the specifics in the program, one possible starting point is illustrated in Figure 3 where the focus groups comprise pillars that link research with the public and animal health agencies providing a two way conduit of information and ideas.

### Scientific Project Management

Successful programs in scientific research depend on translating ideas into scientific results. To foster successful projects at the CPHR implementation of effective scientific management dedicated to the highest quality science is mandatory. While the front line of a management team is the Georgian scientist team trained in scientific project management, during the initial phase of implementation of a SSA, support of an international team of collaborating scientists (with experience in project management) is crucial.

### Diversifying Scientific Funding

While developing the IP, the POE acknowledged that funding for science is highly competitive and funds are very limited, especially for programs where the Principal Investigator scientists are just beginning to establish their reputations. Thus, the POE will be assisting CPHR management in obtaining funding needed to conduct research outlined in the Strategic Scientific Plan. New ideas, resources, experience, and a nurturing scientific environment are all critical to success. While the experience of entering the competitive fray has much to offer in lessons learned and partnerships made, it can be frustrating. A new facility, like the CPHR, makes it necessary to procure funds for operational needs and seeking funds for the research to be done becomes even more challenging. Fortunately, there are international programs that support international scientific development. The POE is to work with the CPHR and international partners to target the relevant funding agencies.

### Building Blocks for Program Development

The POE continues its work with the Georgian scientists and policy makers to develop a strong program of scientific research. Criteria for success will be the establishment of a portfolio of projects undertaken at the CPHR supporting the overall program. The scientific projects will add value to the CPHR program and aid in development of new initiatives for the Georgian Universities. Tools to be included in the program are quantitative bacteriology, phylogenetic analysis, molecular cloning, quorum sensing, informed biosurveillance, and molecular epidemiology, among others. All serve as a basis for the Focus Groups. The goal is to assemble, from the start, projects where the whole is greater than the sum of the parts. It is important to point out that these tools are building blocks and not what are usually considered to be the tools of science, such as an electron microscope or nucleic acid sequencer, but instead people oriented capabilities that can be utilized to understand a general biological process central to a larger question. These building blocks will be important to the regional scientists in understanding disease outbreaks no matter whether its origins are natural, from laboratory accidents, or from intentional releases of synthetic pathogens.

The IP is intended to serve ongoing efforts and capitalize on unique resources and talents in Georgia to provide support for the One Health Initiative.

In summary, the POE provides a unique resource in assistance and guidance to CPHR management as the struggle for diversification in science research and expansion of their funding base get underway. The extended network of international scientists of the POE offers a useful and flexible means for identifying new sources of funding. The POE can work closely with CPHR management to identify funding sources and assist in procuring funding. International funding partners need to be identified and selected scientific areas of research underway should be showcased to leverage the unique resources of CPHR, Georgia, and the region. The POE members can assist in developing proposals and by communicating the progress being made at the CPHR and ensuring CPHR management is informed of scientific funding trends.

## Lessons Learned and Conclusion

The efforts described here do not represent the first group of experienced senior scientists to commit themselves to helping build scientific programs to improve public and animal health in developing regions (6). While the location and details may be different with each such effort, the lessons learned are more often similar. The scientific questions are not what presents the greatest challenge. Nor is it the availability of modern laboratories and scientific equipment that determines success. The most difficult challenge is to obtain the commitment to build the educational foundation of a strong scientific program and to stay the course for the long term, through the frustrating times of establishing a world-class science program. Building a science program in a developing region requires governments and international funding agencies to work together and to understand that seed funds are critical to support the effort. In Georgia, the CRDF Global and the Shota Rustaveli National Science Foundation are working together to provide modest, but extremely important, funding to support the development of research on infectious diseases in Georgia. This endeavor needs understanding and continued support. Research, by its very nature, deals with the unknown, so results are slow in being realized, and the time it takes to obtain societal applications cannot be predicted and described with milestones incorporated into governmental plans. It is a challenge for scientist to put the value of research in terms to government decision makers that they appreciate and can utilize effectively in making their decisions.

The POE has always understood this conundrum and especially that the most important element of building an effective science program is educating and training young, creative scientists and to provide them with the opportunity to do their science in their home country. Considering the need for an educated workforce to fill jobs in the region that require a scientific expertise and to keep these young people focused on solving local problems of public and animal health, the challenge is huge. Many developing countries have not emphasized the need for education in the sciences and technical fields. Too often, they have seen this as an opportunity for their young people to leave the country to gain an education and find jobs elsewhere. International scientists need to help local governments build programs that are focused on solving local problems and contributing to the local economy and providing a reason for young scientists to stay involved at home.

Collaborative science is all about how to do the next good experiment, and it is also all about how to build trust and working relationships, with each other and with those in charge of seeing the program that is being developed has a future for the next generation of young scientists. Decision makers are beginning to recognize what enhancing science in the region can mean for economic development. Scientists need to do a better job in providing decision makers with facts as to how supporting a developing science program translates into lives saved and money well spent.

Another important lesson learned is that it is critical to step back on occasion to assess the appropriate course of action to develop science in a given region. The way western, namely, United States and European, scientists tackle problems may not always be the best for solving problems in the developing world. It is important to listen and understand that your approach may not always be the best for improving science and it needs to be adjusted to the situation and the time the work is ongoing. To enforce a singular point of view may be more harmful to the good relationships that are needed to do good science.

## Security

Because of the threat of bioterrorism, building an international science program dealing with infectious diseases with the goal of promoting threat reduction and global health security is, by its nature, a security challenge. Infectious agents have been and are likely to be used in the future by one people to do harm to another. In addition, concern for accidental exposure of a population to a pathogen, while scientific experiments are being conducted, cannot be ignored, even though modern laboratories and biosafety methods are coupled with aggressive training and have significantly reduced risk of accidental exposures.

An extremely difficult issue when trying to build scientific programs to study especially dangerous pathogens is whether acquiring fundamental knowledge of the mechanisms of action of pathogens represents a security risk. This is a question being grappled with by the research community around the world (7). While there is concern that fundamental knowledge can aid in nefarious development of a biological agent, the practicality is low. The biology of pathogenesis is so complex and modern methods of biological research have yet to match the capabilities of nature. So the benefits gained from knowing the workings of a pathogen and dealing with what nature is developing far outweigh potential negative consequences. The research is ongoing in the developed world, with participation of international students in some laboratories, and the results are publically available in the open literature. As a general statement, it can be said that research in the developing world needs to take advantage of publicly available knowledge and apply it to local challenges to improve the health of those people who need it the most. The portfolio of research that can be conducted in these developing laboratories needs to be allowed to match what is being done under approved regulations around the world and reported in the open literature. The scientific method and tools needed to acquire the knowledge are already at their disposal in the literature, on the internet and often when they are trained overseas, and to attempt to restrict participation in research based solely on country of origin would be misdirected.

## Science Education

After the dissolution of the Soviet Union, education, particularly in the republics outside of Russia itself, suffered greatly. As Georgia, like other former Soviet Union republics began to pull itself out of the turmoil left during the post-Soviet struggle and as its educational institutes began to recover, science was not a priority in the education curriculae. The focus was more on those elements of an education that could immediately stabilize their economies. Education focused on where the jobs were and science jobs are not to be found in the post-Soviet republics. Those Georgians wishing to pursue careers in science were most likely to leave the country, to find science jobs elsewhere. The remaining scientists were those whose training was provided during Soviet times and this was mainly in Russia, along with the few younger students who were trained by these senior scientists. These scientists were trained to support centrally directed science programs with little opportunity for individual investigator driven, hypothesis-based research. It was difficult for a young scientist to become a leader of a research project. To build a science program in these countries without addressing the long term educational elements needed to train young scientists as creative thinkers is essentially a futile exercise. While a strong science education is important, it should be coupled with a place in the local economy for science based jobs. The public and animal health sector, together with support for research at the Universities to develop biotechnologies that help the local economy, is paramount for maintaining sustainable science.

Critical to the POE recommendations both with the SSA and the IP was building a new partnership with Georgian educational institutions. Traditionally, science in these countries during the Soviet times at research institutes was driven by directives from above and success was measured in the number of samples analyzed together with the analyses performed. Epidemiological analysis of the data was excellent, but for most scientists, discovery of fundamental new knowledge about the nature of the pathogens was not promoted. Samples were routinely taken to Russia for these more advanced studies. It was not that Soviet and post-Soviet scientists in the republics lacked the skills to perform hypothesis-driven research, they lacked the motivation. This approach to science has not gone away with the dissolution of the Soviet Union. Coupled with the explosive developments in life sciences around the rest of the world, this mentality has left post-Soviet scientist in the republics with a lack of confidence that they can compete for the funds necessary to carry out relevant research. Most support for research in these countries still comes from international sources because the local governments are focused on other elements of their economy. As the realization that long-term economic growth of the region depends on investment in education in science and technology, the struggle to catch up seems insurmountable, Added to this has been the increasingly competitive nature of generating research funding throughout the community of scientists. To counter this feeling of being behind and having an impossible mission to catch up, the POE recommended a research program that would be uniquely theirs and would focus on ecological problems in their own neighborhood with factors that they uniquely could study and understand, but in turn is of global importance. The long list of newly emerging infectious diseases that come from local regions is evident that these problems are of global importance. Again the POE emphasized that for this program to be a success, there is a need to train young country scientists on how to generate creative new ideas on how to solve important problems. Such training would involve working closely with highly successful scientists from around the world not for just a short period of time, but long enough to learn what it means to come up with a hypothesis and design an approach to test it. There needs to be provided seed money to support the effort of these newly trained scientists to succeed or fail with their own hypotheses. The recommendation is a two-part program of international training along with support to place successful international scientists at the CPHR on sabbatical and work at the Lugar Center along with young Georgian scientists. These types of collaborations bring long-term relationships to be applied to any new infectious disease outbreak that might occur.

Improving science education in the post-Soviet republics remains a long-term goal and can be achieved with the help of international scientists. However, it cannot move forward without a commitment from the local government. The POE supports the goals of international agencies working to help the Georgian government improve education in the sciences by working with organizations like the Millenium Challenge Corporation (8) and the European Union. The POE University training program has been working with the Fogarty International Center of the National Institutes of Health, as well as European Union organizations, to provide support for Georgian students to work in international laboratories and also maintain affiliation with their home institution. This training includes at least a year spent working in the international laboratory. A major requirement is that the student develops and submits a research proposal for a project to be conducted at home in support of the global health security.

## Collaborative Science

Collaborative science in the developing world is not the same as that in Europe or the West. In a setting where science is well developed, collaborative science is working together of research teams with different, but supportive capabilities to address and solve a question that neither group alone can accomplish. In the developing world, collaborative science with participation of international experts must take on a much more inclusive role. The goal is to promote development of modern science for individuals in the host country, not for the financial gain or career development of the international scientist. This is a particularly sensitive issue for both host governments and for host scientists. And if the situation is not absolutely clear, it leads to loss of trust on the part of those whom the program intends to aid. International collaborating scientists must be sure to focus on benefit to the participating scientists in the developing region to ensure a positive impact on science in these regions. This includes appropriate authorship on manuscripts, export of research materials back to western or European laboratories, or hiring away talented young students needed to sustain the program of the developing countries instead of developing mutual partnerships between laboratories. It is particularly difficult for international scientists to remain behind the scenes when on contract by international agencies and their contract depends on showing evidence of their work on the given project. However, this fact is not lost to host country participants and it makes them question the motivation of the international effort. The long-term benefit to science of genuine and full partnership for the sake of science is much more important than any short-term benefit to an individual or corporation.

## Mission Restrictions

The mission space of most funding agencies does not allow for the support of all aspects of a program needed in developing countries to make their mission a success. In regions like the former Soviet Union republics where support of science has been lacking for so many years, building a successful program that can achieve health security and compete for funding in the modern world requires a complete redevelopment of the education system and patient financial support, while a critical mass of scientists can come together to generate new understanding of scientific research relevant to the region. In the area of infectious diseases, this is particularly difficult, not because of the science itself being complex which of course it is, but because of politics. It is important that the local government is a stakeholder in the science program and is committed to see it succeed.

As discussed earlier, working on issues that impact global health security in developing countries presents funding agencies with some difficult problems. While the goal of any program dealing with health security and infectious disease may be clear to the scientists involved, assembling components to make a sustainable meaningful effort is a challenge. It takes more than building laboratories and supplying modern equipment and training in the operations of the systems. It takes building an understanding and commitment to address what is needed. Because of the dynamic nature of infectious agents, whether they originate in nature or from nefarious activity, it requires creative research to be able to respond in a timely manner to contain spread of infection, save lives, and prevent economic disaster. Research at the local level is of prime importance. That research must be supported by funding agencies led by a host government that understands the need for patience in developing a capacity for research on infectious disease.

While the overall goal is to support threat reduction and global health security through science, programs usually operate within their own guidelines that may limit the possibility of achieving the larger goals. Focusing on one part of the problem leaves institutions having to piece together support to maintain facilities, trained scientists, and program goals. Nature does not work from a list of select agents. In the modern world, potential bioterrorists are not restricted to a “Cold War” list of agents to inflict damage to their enemy. Threat reduction in the developing world and globally comes not from restricting scientists to a narrow definition of understanding disease and devising the means for saving lives, but it comes from attacking those problems impacting the health of people in their own neighborhood with creative solution and working to prevent similar outbreaks elsewhere and in the future. Understanding the mechanisms of action of any one pathogen helps in the understanding of all such agents. Next generation nucleic acid sequencing technologies have shown how complex the microbial community really is, but there is yet to be unraveled the overall system of how these agents have come to be, how they operate, and where they are going. The list of ecological factors that shape these outbreaks represents a hugely complex system and ever changing. Understanding these processes needs to start locally and expand globally and will allow us to deal with immediate outbreaks and predict future events.

The ISAC and POE have worked to support development of a relevant and contemporary science program at the CPHR in the Republic of Georgia for ~3 years. The work is far from completed. The POE effort has succeeded in stimulating new scientific ideas on the part of Georgian scientists and has introduced them to new potential collaborators. The work accomplished to date has also challenged the Georgian scientists to consider those issues faced by their country in a new dimension and they have learned how to develop research proposals with themselves as principal investigators. They have joined the community of scientists who are finding universally, that funding for good ideas is hard to find, and the search is frustrating. Workshops on important topics have provided focal points for scientific development in the region and have brought together international experts with excellent discussion and new ideas for projects, none of which have yet been funded but the potential (and hope) remains.

The experience of the POE has proven that there is no shortage of excellent scientists and agencies dedicated to building scientific capacity in developing countries focused on the improvement of public and animal health. All are committed to making a difference, but the agencies operate within the confines of their own missions, and effective integration of these well-intentioned efforts is very much needed.

A key truism is that one cannot predict where the next good idea will come from. And that is certainly is the case in addressing the threat of infectious disease. It is the human component of the equation that matters most and makes the reward of building science programs in the developing world a certainty. It is an investment in the future.

Over the 3 years, the POE has worked diligently to promote research and research capacity for gaining understanding of pathogens in this corner of the world, nature has conducted trillions upon trillions of “experiments” to develop new versions of agents and to challenge life on our planet, all while the world has become a less stable place.

## Ethics Statement

Our study was carried out in accordance with the rules set forth in the Federal Advisory Committee Act of 1972. All members of the Panel of Experts were consultants to the Defense Threat Reduction Agency.

## Conflict of Interest Statement

The authors declare that the research was conducted in the absence of any commercial or financial relationships that could be construed as a potential conflict of interest.
